# Investigating the shared genetic basis and causal relationships between mucosa-associated lymphoid tissue inflammation and psychiatric disorders

**DOI:** 10.3389/fpsyt.2024.1379922

**Published:** 2024-04-29

**Authors:** Andrea N. Georgiou, Konstantinos Voskarides, Panos Zanos, Andreas Chatzittofis

**Affiliations:** ^1^ Department of Psychology, University of Cyprus, Nicosia, Cyprus; ^2^ Department of Basic and Clinical Sciences, University of Nicosia Medical School, Nicosia, Cyprus; ^3^ School of Veterinary Medicine, University of Nicosia, Nicosia, Cyprus; ^4^ Medical School, University of Cyprus, Nicosia, Cyprus; ^5^ Department of Clinical Sciences/Psychiatry, Umeå University, Umeå, Sweden

**Keywords:** tonsillectomy, appendectomy, appendicitis, mucosa-associated lymphoid tissue inflammation, psychiatric disorders, Mendelian randomization

## Abstract

**Background:**

Chronic and acute inflammation of the mucosa-associated lymphoid tissue have been positively linked to the development of psychiatric disorders in observational studies. However, it remains unclear whether this association is causal. In the present study, we investigated this association, using as proxies genetically predicted tonsillectomy, appendectomy and appendicitis on psychiatric disorders including major depressive disorder (MDD), schizophrenia (SCZ), bipolar depression (BD) and anxiety (ANX) via a two-sample Mendelian randomization (MR) analysis.

**Methods:**

Genetic association summary statistics for tonsillectomy, appendectomy and appendicitis were sourced from FinnGen Consortium, comprising data from 342,000 participants. Genetic correlations between all exposures and outcome were calculated with Linkage Disequilibrium Score (LDSC) Regression analysis. MR estimates were then calculated to assess their impact on the risk of developing psychiatric disorders. Sensitivity analysis was employed to test for any directional pleiotropy.

**Results:**

Our results suggest that there is no direct causal association between tonsillectomy, appendectomy or appendicitis with a heightened risk for development of psychiatric disorders. The robustness of the results of the main MR analysis was further confirmed with additional sensitivity analyses. However, a moderate inverse genetic correlation was observed between tonsillectomy and MDD traits (r_g_=-0.39, p-value (P)=7.5x10^-5^).

**Conclusion:**

Our findings provide, for the first time, evidence that there is no causal association between tonsillectomy or appendectomy on subsequent vulnerability of developing psychiatric disorders. Future studies using larger sample size GWAS should focus on unraveling the confounding factors and mediators to investigate this relationship further.

## 1 Introduction

Chronic inflammation is hypothesized to be an underlying pathophysiological risk factor for the development of psychiatric disorders, including, anxiety (ANX), major depressive disorder (MDD) and schizophrenia (SCZ) (1, 2). There are several indications for an association between inflammation and increased susceptibility for the development of psychiatric disorders (3). Through activation of microglia and astrocytes, inflammation is moderated by the immune system through a number of inflammation-related factors including cytokines (4). These neuroinflammatory processes are believed to disrupt neurotransmitter signaling, synaptic plasticity, and neuronal function, thereby contributing to the pathogenesis of disorders such as obsessive-compulsive disorder (OCD), MDD and SCZ (5–8). Brain structure and function alternations have also been reported in individuals with chronic inflammation and psychiatric disorders, suggesting a neural basis related to inflammation for the observed behavioral and affective symptoms (9, 10). A meta-analysis showed that drugs targeting cytokines treated psychiatric disorders in a subset of patients with chronic inflammation (11). However, studies investigating the anti-inflammatory effects of psychotropic drugs had inconsistent findings (4, 12).

Mendelian randomization (MR) has gained attention as a robust method to infer causal association while addressing some of the inherent limitations of observational studies. This methodology can be utilized to investigate the association between outcomes of chronic and acute inflammation with the propensity of developing psychiatric disorders. Tonsillectomy and acute appendicitis are used, respectively, as proxies for chronic and acute inflammation within the mucosa-associated lymphoid tissue (13). A number of studies examined the relationship of both tonsillectomy and acute appendicitis with the risk for developing psychiatric diseases. For example, a Swedish population-based cohort study indicated that individuals exposed to childhood appendectomy had an increased relative risk of developing mood and anxiety disorders, while this association was not observed in individuals with conservatively-treated childhood appendicitis leaving the appendix intact (14). Streptococcus is considered amongst the most common bacterial infections causing tonsillitis (15); however, tonsillectomy has not been reported to successfully prevent the development of pediatric autoimmune neuropsychiatric disorders associated with streptococcal infection (PANDAS) (16, 17). Therefore, the positive association between tonsillectomy and the development of PANDAS that has been reported in case studies might in fact be influenced by the use of postoperative medication, and is currently not supported by the results of larger-scale studies (18).

Importantly, Isung et al., 2019, investigated a Swedish cohort, including 210,686 individuals exposed to tonsillectomy, 86,928 individuals exposed to acute appendectomy, 317,214 clusters of siblings discordant for tonsillectomy, and 160,079 sibling clusters discordant for appendectomy. Their findings indicated that persistent inflammation of mucosa-associated lymphoid tissue is associated with an increased risk for the development of psychiatric disorders. OCD, Tourette Disorder, autism spectrum disorder, attention-deficit/hyperactivity disorder, bipolar disorder, major depression disorder and other mood disorders, generalized anxiety disorder, social anxiety disorder, agoraphobia and substance use disorders were associated with tonsillectomy, The same disorders with the addition of anorexia nervosa but not autism spectrum disorder nor agoraphobia were associated with acute appendicitis (13). Possible mechanisms might include an increase in pro-inflammatory cytokines by innate immune cells in response to the resulting tonsillitis and appendicitis. In turn, cytokines act within the brain to induce symptoms ranging from impaired attention and irritability to anhedonia and disturbed sleep patterns (19, 20).

Observational studies have their limitations such as the influence of confounding factors such as age, gender, and antibiotic exposure. Mendelian randomization has gained attention as a robust approach for inferring causality. It achieves this by utilizing genetic variants as instrumental variables for the exposure under investigation. These genetic variants are characterized by random allocation of alleles, ensuring they are not influenced by reverse causation and possible confounders (21). The aim of the present study was to perform a two-sample MR analysis to investigate the exact association between genetically predicted tonsillectomy, appendicitis and appendectomy with the risk of developing depressive disorder, schizophrenia, bipolar depression and anxiety.

## 2 Materials and methods

**Figure 1:**
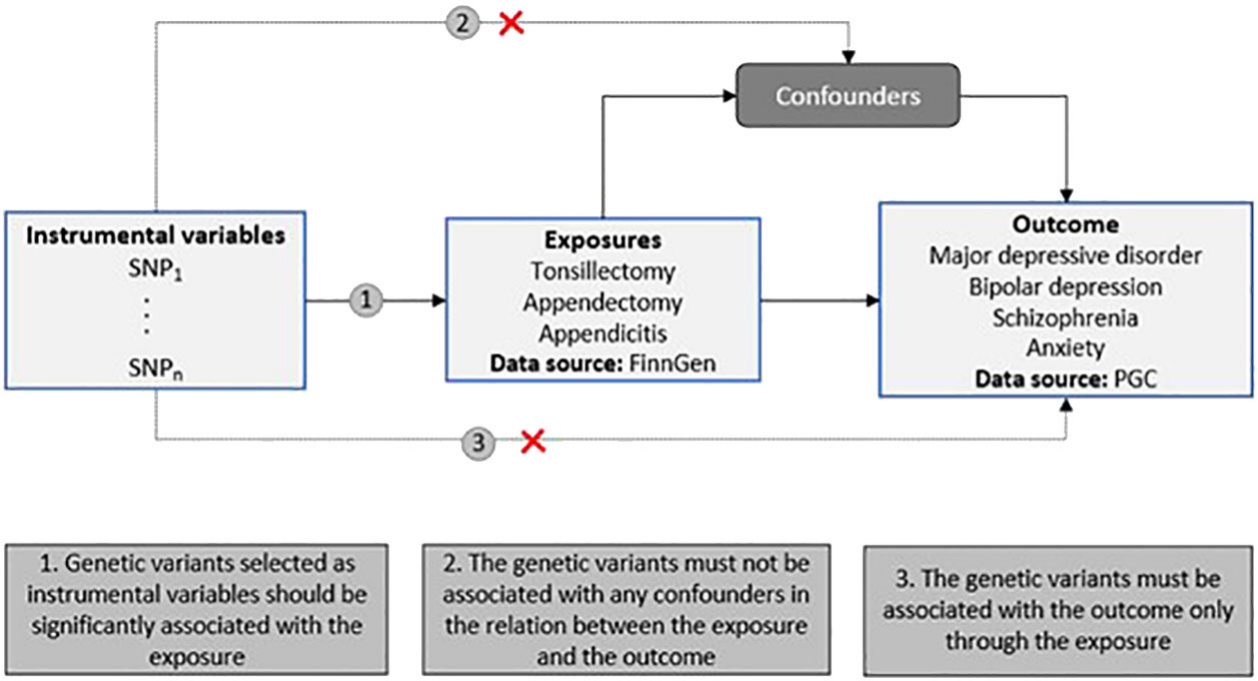
Schematic representation of our two-sample MR study design. The diagram includes the three MR assumptions and the data sources used in the present study for both the exposures and the outcomes (PGC, Psychiatric Genomics Consortium).

We conducted a two-sample MR study to investigate the causal relationship between two risk factors (tonsillectomy, appendectomy and appendicitis (yes *vs* no)) and psychiatric disorders (i.e., major depressive disorder (MDD), schizophrenia (SCZ), bipolar depression (BD) and anxiety (ANX). SNPs associated with the two risk factors were utilized as the instrumental variables (IVs) (**Figure 1**).

### 2.1 Data source and election of IVs

We used summary-level data for tonsillectomy and appendicitis from the FinnGen GWAS Consortium release 8 (R8). Summary association estimates for genome widely associated SNPs (*p*-value (P)<5 x 10^-8^) were obtained in up to 24,544 cases and 317,955 controls for tonsillectomy, up to 23,191 cases and 319,308 controls for appendectomy and in up to 27,523 cases and 314,763 controls for appendicitis (22). To retain the independent IVs, SNPs were clumped and discarded at linkage disequilibrium r^2^ < 0.001 within a 10,000 kb window, which was based on European ancestry reference data from the 1000 Genomes Project.

Genetic association summary statistics for major depressive disorder, schizophrenia, bipolar depression and anxiety were retrieved from the four most recent and largest in terms of participants GWAS meta-analysis studies (23–26) (**Table S1**). The substantial participant numbers contributed to the enhanced statistical power of the study. All selected GWAS from the Finngen Biobank obtained ethical approval from FinnGen Steering Committee, and individuals provided informed consent.

### 2.2 Genetic correlation analysis

Genetic correlation (r_g_) analysis quantifies the shared genetic architecture between two traits, independent of environmental confounders. We utilized linkage disequilibrium score regression (LDSC) to estimate the global genetic correlations between tonsillectomy, appendectomy and appendicitis using GWAS summary data. LDSC leverages GWAS effect size estimates for each locus, encompassing the effects of all variants in linkage disequilibrium (LD) with that locus (27, 28). We considered a Bonferroni-corrected significance threshold of P<0.004 (0.05/12) for statistical significance.

### 2.3 Mendelian randomization analysis

The inverse variance weighted (IVW) method was used as the main MR analysis and the weighted median (WM) and MR-Egger methods as sensitivity analyses. The three methods were based on three different assumptions: (1) the selected SNPs are associated with the exposure; (2) the selected SNPs are not associated with confounders; and (3) the selected SNPs are associated with the outcome exclusively through their effect on the exposure (29). MR-PRESSO and Cochran’s Q statistics were used to evaluate pleiotropy and heterogeneity, respectively. The F-statistic was calculated to evaluate genetic instrument strength. MR odds ratio (OR) for the two exposures for which we obtained a statistical power of 80%, setting the alpha level at 0.05 and using the variance explained for each exposure (R2), was calculated. All MR analyses were conducted using the R (version 4.3.0) statistical environment and TwoSampleMR package (30).

## 3 Results

### 3.1 Genetic correlation

**Figure 2:**
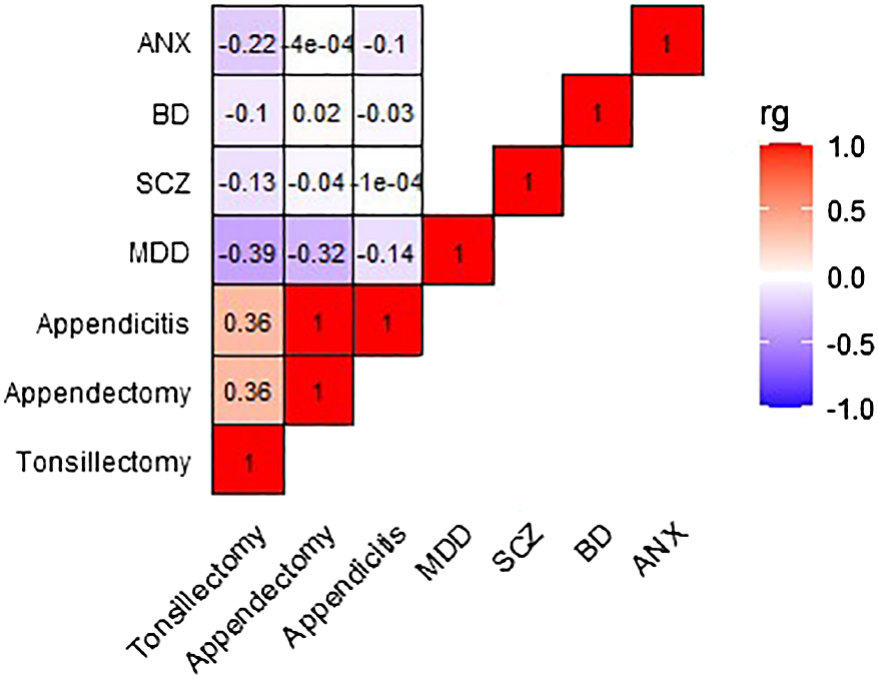
Heatmap depicting the genetic correlation between tonsillectomy, appendectomy, and appendicitis, and MDD, SCZ, BD, and ANX. Genetic correlation was computed using LDSC analysis. rg, genetic correlation; LDSC, Linkage Disequilibrium Score Regression; MDD, major depressive disorder; SCZ, schizophrenia; BD, bipolar disorder; ANX, anxiety.

**Figure 2** and **Table S5** present the genetic correlation results between exposures and outcomes, with tonsillectomy exhibiting a weak to moderate inverse correlation with all four outcomes. Only the genetic correlation between tonsillectomy and MDD surpasses multiple testing correction (MDD: r_g_=-0.39, P=7.5x10^-5^, SCZ: r_g_=-0.13, P=0.01, BD: r_g_=-0.10, P=0.03, ANX: r_g_=-0.22, P=0.006).

### 3.2 Mendelian randomization

**Figure 3:**
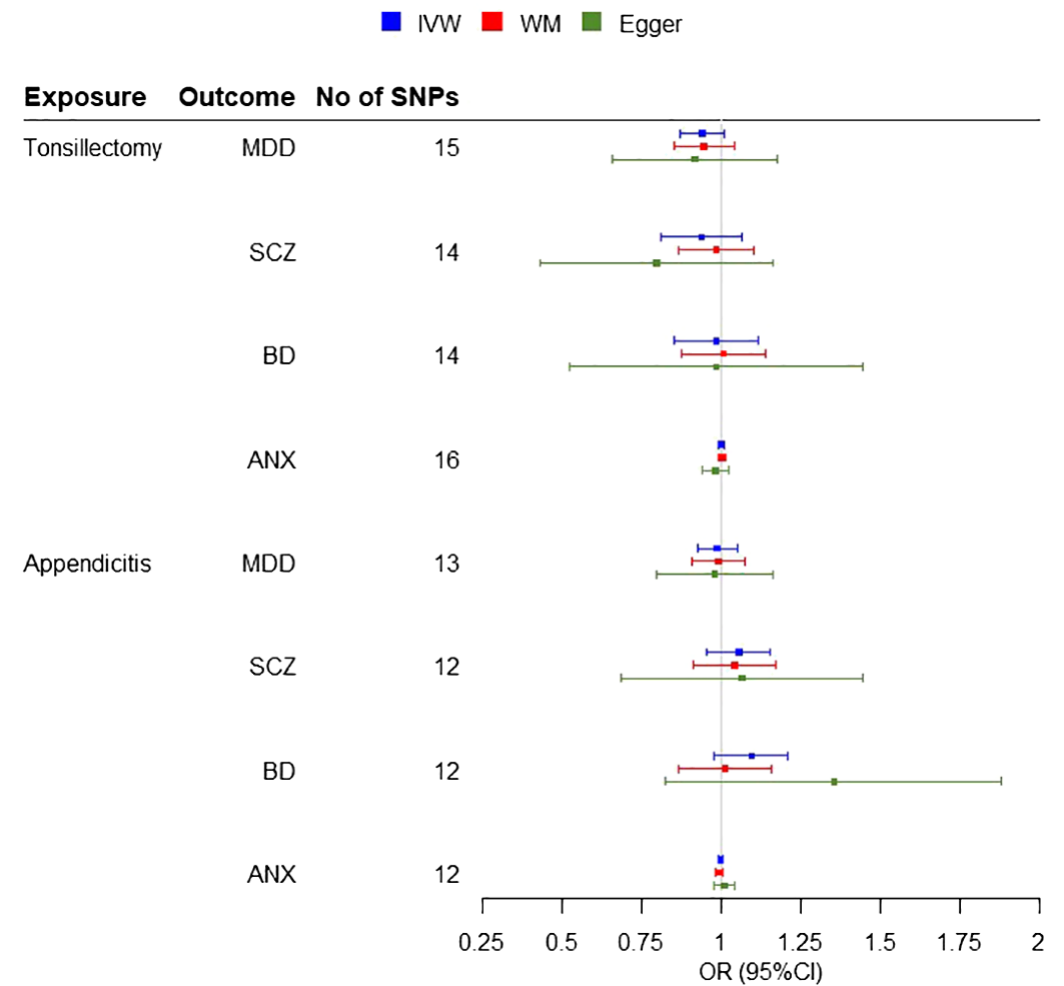
Forest plots for the MR Inverse variance weighted (IVW) analysis and sensitivity results. Investigating the association of genetic susceptibility to tonsillectomy and appendicitis with a number of psychiatric diseases (MDD, Major Depressive Disorder; SCZ, Schizophrenia; BD, Bipolar disorder; ANX, Anxiety disorder; OR, odds ratio; CI, confidence interval; WM, Weighted Median).

A two-sample MR analysis was conducted, without overlap between the exposure and outcome samples, including three potential risk factors (tonsillectomy, appendectomy and appendicitis) for four psychiatric diseases. IVW Cochran’s Q analysis of *p* < 0.05, indicates that there is heterogeneity between SNPs, therefore IVW random effect model was used as a main analysis method. MR analysis did not support any causal association between any exposure and outcome (**Figure 2**). We observed non-significant estimates for tonsillectomy on MDD (IVW OR = 0.94, 95% confidence interval (CI): 0.87 to 1.01, P=0.08), SCZ (IVW OR = 0.93, 95% CI: 0.83 to 1.07, P=0.29), BD (IVW OR = 0.97, 95% CI: 0.85 to 1.12, P=0.70) and ANX (IVW OR = 1, 95% CI: 0.99 to 1.01, 0.99, P=0.99) and for appendicitis on MDD (IVW OR = 0.98, 95% CI: 0.92 to 1.05, P=0.64), SCZ (IVW OR = 1.05, 95% CI: 0.96 to 1.15, P=0.31), BD (IVW OR = 1.09, 95% CI: 0.98 to 1.21, P=0.12) and ANX (IVW OR = 1, 95% CI: 0.99 to 1.01, P=0.57) (**Figure 3**). No evidence of association was observed between appendectomy and any of the psychiatric disorders either (MDD (IVW OR = 0.95, 95% CI: 0.82 to 1.09, P=0.44), SCZ (IVW OR = 0.99, 95% CI: 0.85 to 1.17, P=0.96), BD (IVW OR = 0.98, 95% CI: 0.82 to 1.16, P=0.80)).

In the sensitivity analysis, there was no evidence of pleiotropy (MR–Egger intercept *p* > 0.05, MR–PRESSO Global Test *p* > 0.05), with the exception of the tonsillectomy and SCZ association (MR–Egger intercept *P* = 0.28, MR–PRESSO Global Test *P* < 0.001) The estimates provided by sensitivity analyses were consistent with the main findings. High heterogeneity was observed for all associations between tonsillectomy and psychiatric diseases (Cochran Q >31.25, *P* < 0.05), whereas no heterogeneity was observed for appendicitis or appendectomy and psychiatric diseases (Cochran Q < 12, *P* > 0.05) (**Table 1**).

**Table 1:** Results of inverse variance-weighted MR and sensitivity analysis for exposures and risk of outcomes.

Exposure	Outcome	No SNPs	IVW: OR (95%CI)	MR-Egger: OR (95%CI)	MR-PRESSO p-value	Cochran’s Q p-value
**Tonsillectomy**	MDD	15	0.94 (0.87 - 1.01)	0.88 (0.66 - 1.18)	0.31	0.29
	SCZ	14	0.93 (0.81 - 1.07)	0.71 (0.43 - 1.16)	<0.001	<0.001
	BD	14	0.97 (0.85 - 1.12)	0.87 (0.52 - 1.45)	0.001	0.001
	ANX	16	1.00 (0.99 - 1.01)	0.98 (0.94 - 1.02)	0.003	0.008
**Appendectomy**	MDD	3	0.95 (0.82 - 1.09)	1.08 (0.40 - 2.05)	NA	0.73
	SCZ	3	0.99 (0.85 - 1.17)	0.65 (0.02 - 179)	NA	0.41
	BD	3	0.98 (0.82 -1.16)	2.8 (0.03 - 270)	NA	0.89
**Appendicitis**	MDD	13	0.98 (0.92 - 1.05)	0.96 (0.80 - 1.16)	0.69	0.6
	SCZ	12	1.05 (0.96 - 1.15)	0.99 (0.69 - 1.44)	0.44	0.4
	BD	12	1.09 (0.98 - 1.21)	1.25 (0.83 - 1.88)	0.32	0.33
	ANX	12	1.00 (0.99 - 1.01)	1.01 (0.98 - 1.04)	0.98	0.98

CI, confidence interval; IVW, inverse variance-weighted; MR, Mendelian randomization; SNP, single nucleotide polymorphism.NA, Not applicable.

All retained SNPs exhibited F-statistics greater than 10, indicating a strong association between the instrumental variables and tonsillectomy, appendicitis and appendectomy (**Tables S2**-**S4**). Based on sample sizes of 342,499 and 342,486 individuals for tonsillectomy and appendicitis, respectively, and setting significant level alpha at 0.05, our MR study had 80% statistical power to detect effects on psychiatric diseases of an OR ranging from 1.25 to 1.30 per unit of each exposure. Appendectomy only comprises by three IVs and hence the power to detect any association for this exposure is considerably smaller (OR > 1.7).

## 4 Discussion

Leveraging data from the largest available GWAS consortia, this MR study shows, for the first time, no evidence for a direct causal association between genetically predicted tonsillectomy and appendicitis and heightened risk for developing psychiatric disorders. Our findings were consistent using different MR sensitivity methods testing for violations of assumptions. There is evidence for a moderate inverse correlation between the genetic architecture of tonsillectomy and MDD. Therefore, individuals with a genetic predisposition to tonsillectomy may have a lower genetic risk for major depression, and vice versa. However, the above does not imply a causal relationship between tonsillectomy and major depression. Other factors, such as environmental influences and shared biological pathways, may also contribute to both traits. Our findings contribute towards the current body of evidence and highlights that the association between tonsillectomy or appendicitis on MDD, SCZ, bipolar depression and anxiety is more likely to be indirect and not of a genetic cause. Prior research concerning this relationship is limited, and only observational studies exist to report such associations. Therefore, our study provides critical insights by addressing this research gap.

The tonsils and appendix are part of the mucosa-associated lymphoid tissue, which provides a defensive barrier against pathogens. Surgical procedures to remove these organs in early childhood or adolescence, may cause long-term alteration in immune function, and in turn, they influence the risk for developing chronic diseases (31). Although chronic inflammation is known to be a risk factor for psychiatric diseases, the exact pathophysiology mechanism still remains unknown.

Much of the existing data regarding the role of immune dysfunction in neurodegenerative diseases yields conflicting or inconclusive results, probably due to the methodological variability across studies and in some cases due to small sample sizes. For example, patient groups in these studies are highly heterogeneous, which can affect the results; since the immune system varies between males and females and its response is more robust in younger than in older groups (32, 33). Another MR study explored the causal association between 731 immune cell signatures and SCZ risk, revealing that only four immunophenotypes exhibited a significant association with SCZ (34). In addition, patients undergoing tonsillectomy have often been exposed to prior antibiotics treatment (35). By utilizing MR in our study, we avoid bias due to residual confounders such as age, gender or even exposure to antibiotics that exists in observational studies. In addition, circulatory inflammatory cytokine levels are significantly elevated in patients with psychotic and affective disorders, with significant heterogeneity in study findings (36). Therefore, mediators such as cytokines probably exist within the pathway from the investigated exposure to outcome. A possible explanation of our results could be the moderating effects of gut microbiota. The microbiota-gut-brain axis, has an important role in homeostasis and regulation of peripheral and central inflammation with important effects on the development of psychiatric disorders (5). Additionally, epigenetic modifications are likely to be implicated in the development of psychiatric phenotypes. The impact of childhood adversity has been highlighted in the development of psychiatric disorders through the modification of the neuroendocrine and immune systems (37, 38).

The present study is not without limitations. Specifically, the inability to perform mediation analysis owing to the limited number of instrumental variables for cytokines. Another limitation is regarding the generalizability of our findings to non-European populations as that GWAS data that were used for our study have all derived from individuals of European ancestry. It is important to mention that our MR study can confidently exclude effects larger than an OR of 1.3, and hence smaller effects cannot be excluded. Therefore, future well-powered MR studies are needed to investigate these effects.

In conclusion, our study uses MR analysis to explore the potential causal relationship between tonsillectomy and appendicitis and the risk of developing psychiatric disorders. It provides robust evidence, for the first time, that the previous reported associations are probably not of a genetic cause (13, 14). These findings challenge previous assumptions and highlight the importance of considering alternative explanations for the observed associations in observational studies. The MR design has the strength for reducing bias from environmental factors when assessing associations to infer causal effects and is not affected by reverse causality. In addition, the study identifies a moderate inverse correlation between the genetic architecture of tonsillectomy and major depression, suggesting a potential genetic link between those two traits. While this does not imply a causal relationship, it prompts further investigation into the shared genetic factors underlying tonsillectomy and MDD. Notably, psychiatric disorders are highly heterogenous with genetic, epigenetic, and environmental factors underlying psychopathology that can influence the outcome phenotype. Thus, it is important, in the era of precision psychiatry to further investigate possible biomarkers that hold promise in elucidating pathophysiological mechanisms and applying personalized treatment.

## Data availability statement

The original contributions presented in the study are included in the article/**Supplementary Material**. Further inquiries can be directed to the corresponding author.

## Ethics statement

Ethical approval was not required for the study involving humans in accordance with the local legislation and institutional requirements. Written informed consent to participate in this study was not required from the participants or the participants’ legal guardians/next of kin in accordance with the national legislation and the institutional requirements.

## Author contributions

AG: Writing – original draft, Writing – review & editing, Data curation, Methodology. KV: Writing – original draft, Writing – review & editing, Conceptualization. PZ: Writing – original draft, Writing – review & editing. AC: Writing – original draft, Writing – review & editing, Conceptualization, Supervision.

## References

[1] Fond G. Inflammation in psychiatric disorders. Eur Psychiatry. (2020) 29:551–2. doi: 10.1016/j.eurpsy.2014.09.347

[2] Jeon SW Yoon HK Kim YK. Role of inflammation in psychiatric disorders. Adv Exp Med Biol. (2019) 1192:491–501. doi: 10.1007/978-981-32-9721-0_24 31705510

[3] Kivimäki M Shipley MJ Batty GD Hamer M Akbaraly TN Kumari M. Long-term inflammation increases risk of common mental disorder: a cohort study. Mol Psychiatry. (2014) 19:149–50. doi: 10.1038/mp.2013.35 PMC3903110 23568195

[4] Yuan N Chen Y Xia Y Dai J Liu C. Inflammation-related biomarkers in major psychiatric disorders: a cross-disorder assessment of reproducibility and specificity in 43 meta-analyses. Trans Psychiatry. (2019) 9:233. doi: 10.1038/s41398-019-0570-y PMC6751188 31534116

[5] Kouba BR de Araujo Borba L Borges de Souza P Gil-Mohapel J Rodrigues ALS. Role of inflammatory mechanisms in major depressive disorder: from etiology to potential pharmacological targets. Cells. (2024) 13:423. doi: 10.3390/cells13050423 38474387 PMC10931285

[6] Saccaro LF Schilliger Z Perroud N Piguet C. Inflammation, anxiety, and stress in attention-deficit/hyperactivity disorder. Biomedicines. (2021) 9. doi: 10.3390/biomedicines9101313 PMC8533349 34680430

[7] Vreeland A Calaprice D Or-Geva N Frye RE Agalliu D Lachman HM. Postinfectious inflammation, autoimmunity, and obsessive-compulsive disorder: sydenham chorea, pediatric autoimmune neuropsychiatric disorder associated with streptococcal infection, and pediatric acute-onset neuropsychiatric disorder. Dev Neurosci. (2023) 45:361–74. doi: 10.1159/000534261 37742615

[8] Warren N O'Gorman C Horgan I Weeratunga M Halstead S Moussiopoulou J. Inflammatory cerebrospinal fluid markers in schizophrenia spectrum disorders: A systematic review and meta-analysis of 69 studies with 5710 participants. Schizophr Res. (2024) 266:24–31. doi: 10.1016/j.schres.2024.02.001 38364730

[9] Tanaka M Spekker E Szabó Á Polyák H Vécsei L. Modelling the neurodevelopmental pathogenesis in neuropsychiatric disorders. Bioactive kynurenines and their analogues as neuroprotective agents—in celebration of 80th birthday of Professor Peter Riederer. J Neural Transm. (2022) 129:627–42. doi: 10.1007/s00702-022-02513-5 35624406

[10] Battaglia MR Di Fazio C Battaglia S. Activated Tryptophan-Kynurenine metabolic system in the human brain is associated with learned fear. Front Mol Neurosci. (2023) 16:1217090. doi: 10.3389/fnmol.2023.1217090 37575966 PMC10416643

[11] Kappelmann N Lewis G Dantzer R Jones PB Khandaker GM. Antidepressant activity of anti-cytokine treatment: a systematic review and meta-analysis of clinical trials of chronic inflammatory conditions. Mol Psychiatry. (2018) 23:335–43. doi: 10.1038/mp.2016.167 PMC5794896 27752078

[12] Johnson M Ehlers S Fernell E Hajjari P Wartenberg C Wallerstedt SM. Anti-inflammatory, antibacterial and immunomodulatory treatment in children with symptoms corresponding to the research condition PANS (Pediatric Acute-onset Neuropsychiatric Syndrome): A systematic review. PloS One. (2021) 16:e0253844. doi: 10.1371/journal.pone.0253844 34197525 PMC8248649

[13] Isung J Isomura K Almqvist C Lichtenstein P Larsson H Wester T. Association of chronic and acute inflammation of the mucosa-associated lymphoid tissue with psychiatric disorders and suicidal behavior. Transl Psychiatry. (2019) 9:227. doi: 10.1038/s41398-019-0568-5 31515504 PMC6742630

[14] Ekström LD Ekström H Dal H Kosidou K Gustafsson UO. Childhood appendectomy and adult mental disorders: A population-based cohort study. Depress Anxiety. (2020) 37:1108–17. doi: 10.1002/da.23045 32668089

[15] Barzilai A Miron D Sela S. Etiology and management of acute and recurrent group A streptococcal tonsillitis. Curr Infect Dis Rep. (2001) 3:217–23. doi: 10.1007/s11908-001-0023-6 11384551

[16] Demesh D Virbalas JM Bent JP. The role of tonsillectomy in the treatment of pediatric autoimmune neuropsychiatric disorders associated with streptococcal infections (PANDAS). JAMA Otolaryngol Head Neck Surg. (2015) 141:272–5. doi: 10.1001/jamaoto.2014.3407 25569020

[17] Sigra S Hesselmark E Bejerot S. Treatment of PANDAS and PANS: a systematic review. Neurosci Biobehav Rev. (2018) 86:51–65. doi: 10.1016/j.neubiorev.2018.01.001 29309797

[18] Windfuhr JP. Tonsillectomy remains a questionable option for pediatric autoimmune neuropsychiatric disorders associated with streptococcal infections (PANDAS). GMS Curr Top Otorhinolaryngol Head Neck Surg. (2016) 15:Doc07. doi: 10.3205/cto000134 28025607 PMC5169080

[19] Haroon E Raison CL Miller AH. Psychoneuroimmunology meets neuropsychopharmacology: translational implications of the impact of inflammation on behavior. Neuropsychopharmacology. (2012) 37:137–62. doi: 10.1038/npp.2011.205 PMC3238082 21918508

[20] Pape K Tamouza R Leboyer M Zipp F. Immunoneuropsychiatry - novel perspectives on brain disorders. Nat Rev Neurol. (2019) 15:317–28. doi: 10.1038/s41582-019-0174-4 30988501

[21] Davies NM Holmes MV Smith GD. Reading Mendelian randomisation studies: a guide, glossary, and checklist for clinicians. BMJ. (2018) 362:k601. doi: 10.1136/bmj.k601 30002074 PMC6041728

[22] Kurki MI Karjalainen J Palta P Sipilä TP Kristiansson K Donner KM. FinnGen provides genetic insights from a well-phenotyped isolated population. Nature. (2023) 613:508–18. doi: 10.1038/s41586-022-05473-8 PMC9849126 36653562

[23] Revealing the complex genetic architecture of obsessive-compulsive disorder using meta-analysis. Mol Psychiatry. (2018) 23:1181–8. doi: 10.1038/mp.2017.154 PMC6660151 28761083

[24] Mullins N Ripke S Mattheisen M Trzaskowski M Byrne EM Abdellaoui A. Genome-wide association study of more than 40,000 bipolar disorder cases provides new insights into the underlying biology. Nat Genet. (2021) 53:817–29. doi: 10.1016/j.euroneuro PMC8192451 34002096

[25] Trubetskoy V Forstner AJ O'Connell KS Coombes B Coleman JRI Qiao Z. Mapping genomic loci implicates genes and synaptic biology in schizophrenia. Nature. (2022) 604:502–8. doi: 10.1038/s41588-021-00857-4 PMC9392466 35396580

[26] Wray NR Pardiñas AF Qi T Panagiotaropoulou G Awasthi S Bigdeli TB. Genome-wide association analyses identify 44 risk variants and refine the genetic architecture of major depression. Nat Genet. (2018) 50:668–81. doi: 10.1038/s41586-022-04434-5 PMC5934326 29700475

[27] Ni G Moser G Wray NR Lee SH. Estimation of genetic correlation via linkage disequilibrium score regression and genomic restricted maximum likelihood. Am J Hum Genet. (2018) 102:1185–94. doi: 10.1016/j.ajhg PMC5993419 29754766

[28] Bulik-Sullivan BK Loh PR Finucane HK Ripke S Yang J Patterson N. LD Score regression distinguishes confounding from polygenicity in genome-wide association studies. Nat Genet. (2015) 47:291–5. doi: 10.1038/ng.3211 PMC4495769 25642630

[29] Haycock PC Burgess S Wade KH Bowden J Relton C Davey Smith G. Best (but oft-forgotten) practices: the design, analysis, and interpretation of Mendelian randomization studies. Am J Clin Nutr. (2016) 103:965–78. doi: 10.3945/ajcn.115.118216 PMC4807699 26961927

[30] Hemani G Zheng J Elsworth B Wade KH Haberland V Baird D. The MR-Base platform supports systematic causal inference across the human phenome. Elife. (2018) 7. doi: 10.7554/eLife.34408 PMC5976434 29846171

[31] Liu B Fang F Ye W Wirdefeldt K. Appendectomy, tonsillectomy and parkinson’s disease risk: A Swedish register-based study. Front Neurol. (2020) 11:510. doi: 10.3389/fneur.2020.00510 32595591 PMC7292857

[32] Klein SL Flanagan KL. Sex differences in immune responses. Nat Rev Immunol. (2016) 16:626–38. doi: 10.1038/nri.2016.90 27546235

[33] Montecino-Rodriguez E Berent-Maoz B Dorshkind K. Causes, consequences, and reversal of immune system aging. J Clin Invest. (2013) 123:958–65. doi: 10.1172/JCI64096 PMC3582124 23454758

[34] Wang C Zhu D Zhang D Zuo X Yao L Liu T. Causal role of immune cells in schizophrenia: Mendelian randomization (MR) study. BMC Psychiatry. (2023) 23:590. doi: 10.1186/s12888-023-05081-4 37582716 PMC10428653

[35] Sun W Han X Wu S Yang C. Tonsillectomy and the risk of inflammatory bowel disease: A systematic review and meta-analysis. J Gastroenterol Hepatol. (2016) 31:1085–94. doi: 10.1111/jgh.13273 26678358

[36] Hughes HK Ashwood P. Overlapping evidence of innate immune dysfunction in psychotic and affective disorders. Brain Behavior Immun - Health. (2020) 2:100038. doi: 10.1016/j.bbih.2020.100038 PMC8474635 34589829

[37] Jokinen J Boström AE Dadfar A Ciuculete DM Chatzittofis A Åsberg M. Epigenetic changes in the CRH gene are related to severity of suicide attempt and a general psychiatric risk score in adolescents. EBioMedicine. (2018) 27:123–33. doi: 10.1016/j.ebiom.2017.12.018 PMC5828554 29277323

[38] Yuan M Yang B Rothschild G Mann JJ Sanford LD Tang X. Epigenetic regulation in major depression and other stress-related disorders: molecular mechanisms, clinical relevance and therapeutic potential. Signal Transduct Target Ther. (2023) 8:309. doi: 10.1038/s41392-023-01519-z 37644009 PMC10465587

