# Supplementary material for: Investigating the shared genetic basis and causal relationships between mucosa-associated lymphoid tissue inflammation and psychiatric disorders
Source: Front Psychiatry. 2024 Apr 29;15:1379922. doi: 10.3389/fpsyt.2024.1379922 (PMC11089192; doi:10.3389/fpsyt.2024.1379922)
Supplement: Supplementary file 1 [file Table_1.docx]

**Supplementary Material**

**Table S1:** Data sources of genetic association for the exposures and the outcomes.

| **Trait** | **Cohort/Consortium** | **Sample size** | **First author, Year** |
| --- | --- | --- | --- |
| **Exposure** |  |  |  |
| Tonsillectomy | FinnGen | 24,544 cases/ 317,955 controls | Kurki, 2023 |
| Appendectomy | FinnGen | 23,191 cases/ 319,308 controls | Kurki, 2024 |
| Appendicitis | FinnGen | 27,523 cases / 314,763 controls | Kurki, 2025 |
| **Outcome** |  |  |  |
| Major Depressive Disorder | Psychiatric Genomics Consortium | 135,458 cases/ 344,901 controls | Wray, 2018 |
| Schizophrenia | Psychiatric Genomics Consortium | 53,386 cases/ 77,258 controls | Trubetskoy, 2022 |
| Bipolar disorder | Psychiatric Genomics Consortium | 40,463 cases/ 313,436 controls | Mullins, 2021 |
| Anxiety | Psychiatric Genomics Consortium | 282802 total | Schoeler, 2023 |

**Table S2:** SNP list for exposure to tonsillectomy

| SNP | effect allele | other allele | beta | se | p-value | eaf | exposure | sample size | R2 | F statistic | outcome |
| --- | --- | --- | --- | --- | --- | --- | --- | --- | --- | --- | --- |
| rs1055821 | G | T | 0.14 | 0.02 | 2.36E-09 | 0.96 | Tonsillectomy | 342499 | 0.0001 | 34.36 | MDD |
| rs10849448 | A | G | -0.11 | 0.01 | 3.24E-21 | 0.24 | Tonsillectomy | 342499 | 0.0003 | 89.29 | MDD, BD, SCZ, ANX |
| rs113160481 | A | G | 0.19 | 0.02 | 5.31E-14 | 0.96 | Tonsillectomy | 342499 | 0.0002 | 56.37 | MDD, BD, SCZ, ANX |
| rs11773763 | C | T | -0.06 | 0.01 | 2.73E-09 | 0.69 | Tonsillectomy | 342499 | 0.0001 | 34.95 | MDD |
| rs12128267 | A | G | 0.08 | 0.02 | 2.87E-08 | 0.88 | Tonsillectomy | 342499 | 0.0001 | 30.49 | MDD, BD, SCZ, ANX |
| rs12171069 | G | C | 0.07 | 0.01 | 5.65E-10 | 0.78 | Tonsillectomy | 342499 | 0.0001 | 37.97 | MDD, BD, SCZ, ANX |
| rs1265566 | C | T | 0.07 | 0.01 | 1.17E-10 | 0.39 | Tonsillectomy | 342499 | 0.0001 | 41.36 | MDD, BD, SCZ, ANX |
| rs13214865 | C | T | 0.15 | 0.02 | 2.31E-11 | 0.95 | Tonsillectomy | 342499 | 0.0001 | 44.47 | BD, SCZ, ANX |
| rs174714 | T | C | -0.06 | 0.01 | 2.36E-08 | 0.68 | Tonsillectomy | 342499 | 0.0001 | 30.81 | MDD, BD, SCZ, ANX |
| rs2524035 | A | G | 0.06 | 0.01 | 3.09E-08 | 0.29 | Tonsillectomy | 342499 | 0.0001 | 30.59 | BD, SCZ, ANX |
| rs34767118 | A | G | -0.06 | 0.01 | 2.74E-09 | 0.69 | Tonsillectomy | 342499 | 0.0001 | 34.95 | BD, SCZ, ANX |
| rs3891956 | G | A | -0.07 | 0.01 | 1.41E-09 | 0.78 | Tonsillectomy | 342499 | 0.0001 | 36.23 | MDD, BD, SCZ |
| rs480100 | G | A | -0.13 | 0.02 | 7.02E-10 | 0.06 | Tonsillectomy | 342499 | 0.0001 | 38.02 | MDD, BD, SCZ, ANX |
| rs6533181 | T | G | 0.07 | 0.01 | 1.68E-11 | 0.32 | Tonsillectomy | 342499 | 0.0001 | 45.20 | MDD, BD, SCZ, ANX |
| rs67820526 | G | A | -0.08 | 0.01 | 4.91E-09 | 0.84 | Tonsillectomy | 342499 | 0.0001 | 33.86 | MDD, BD, SCZ, ANX |
| rs572432217 | G | C | 0.16 | 0.03 | 4.86E-09 | 0.97 | Tonsillectomy | 342499 | 0.0001 | 34.13 | MDD |
| rs633862 | T | C | -0.07 | 0.01 | 1.69E-12 | 0.59 | Tonsillectomy | 342499 | 0.0001 | 49.32 | MDD, ANX |
| rs713875 | C | G | 0.10 | 0.01 | 6.44E-26 | 0.52 | Tonsillectomy | 342499 | 0.0003 | 109.97 | MDD, BD, SCZ, ANX |
| rs72635708 | T | C | -0.07 | 0.01 | 7.36E-09 | 0.75 | Tonsillectomy | 342499 | 0.0001 | 33.03 | MDD, BD, SCZ, ANX |
| rs7551487 | A | T | 0.06 | 0.01 | 1.07E-08 | 0.70 | Tonsillectomy | 342499 | 0.0001 | 32.31 | MDD, BD, SCZ, ANX |
| rs77580381 | C | G | 0.15 | 0.03 | 4.10E-08 | 0.97 | Tonsillectomy | 342499 | 0.0001 | 30.00 | MDD, BD, SCZ, ANX |
| rs75820432 | C | T | 0.17 | 0.03 | 4.63E-10 | 0.97 | Tonsillectomy | 342499 | 0.0001 | 38.69 | MDD |
| rs816340 | G | A | 0.07 | 0.01 | 1.33E-09 | 0.73 | Tonsillectomy | 342499 | 0.0001 | 36.31 | MDD, BD, SCZ, ANX |
| rs8176645 | A | T | -0.07 | 0.01 | 1.88E-13 | 0.46 | Tonsillectomy | 342499 | 0.0002 | 53.80 | BD, SCZ |

**Table S3:** SNP list for exposure to appendicitis

| SNP | effect allele | other allele | beta | se | p-value | eaf | exposure | sample size | R2 | F | Outcome |
| --- | --- | --- | --- | --- | --- | --- | --- | --- | --- | --- | --- |
| rs10748784 | G | A | 0.05 | 0.01 | 1.45E-08 | 0.46 | Appendicitis | 342,286 | 0.00009 | 31.88 | MDD, BD |
| rs10849448 | A | G | 0.07 | 0.01 | 1.73E-11 | 0.24 | Appendicitis | 342,286 | 0.00013 | 45.19 | MDD, BD |
| rs114291795 | C | G | -0.09 | 0.02 | 4.55E-08 | 0.92 | Appendicitis | 342,286 | 0.00009 | 29.65 | MDD, BD |
| rs13121924 | A | G | -0.15 | 0.01 | 9.50E-63 | 0.47 | Appendicitis | 342,286 | 0.00081 | 277.50 | MDD, BD |
| rs1497732 | C | T | 0.07 | 0.01 | 6.48E-11 | 0.26 | Appendicitis | 342,286 | 0.00012 | 42.59 | MDD, BD |
| rs17041972 | G | A | 0.08 | 0.01 | 5.55E-10 | 0.88 | Appendicitis | 342,286 | 0.00011 | 38.01 | MDD, BD |
| rs1978995 | G | A | -0.07 | 0.01 | 5.66E-10 | 0.20 | Appendicitis | 342,286 | 0.00011 | 38.40 | MDD, BD |
| rs200540616 | C | T | 0.07 | 0.01 | 3.35E-11 | 0.78 | Appendicitis | 342,286 | 0.00013 | 43.29 | MDD, BD |
| rs201768 | T | C | 0.07 | 0.01 | 7.19E-11 | 0.28 | Appendicitis | 342,286 | 0.00012 | 42.38 | MDD |
| rs2595100 | C | T | 0.08 | 0.01 | 4.25E-17 | 0.61 | Appendicitis | 342,286 | 0.00020 | 69.75 | MDD, BD |
| rs3738182 | G | A | -0.09 | 0.01 | 3.09E-18 | 0.76 | Appendicitis | 342,286 | 0.00022 | 74.73 | MDD, BD |
| rs3850479 | T | C | 0.07 | 0.01 | 2.12E-12 | 0.33 | Appendicitis | 342,286 | 0.00014 | 49.21 | MDD, BD |
| rs73096135 | C | T | 0.14 | 0.02 | 2.48E-08 | 0.97 | Appendicitis | 342,286 | 0.00009 | 30.94 | MDD, SCZ, BD |
| rs9276057 | A | G | -0.05 | 0.01 | 2.19E-08 | 0.44 | Appendicitis | 342,286 | 0.00009 | 31.10 | MDD |
| rs10021629 | G | A | 0.07 | 0.01 | 1.30E-08 | 0.87 | Appendicitis | 342,286 | 0.00009 | 31.91 | SCZ, ANX |
| rs11064157 | A | C | 0.06 | 0.01 | 1.94E-08 | 0.28 | Appendicitis | 342,286 | 0.00009 | 31.49 | SCZ, ANX |
| rs1268337 | A | G | 0.06 | 0.01 | 7.19E-09 | 0.7 | Appendicitis | 342,286 | 0.00010 | 35.50 | SCZ, ANX |
| rs1445132 | G | C | 0.07 | 0.01 | 5.94E-10 | 0.806 | Appendicitis | 342,286 | 0.00014 | 48.09 | SCZ, ANX |
| rs150472801 | G | T | 0.15 | 0.03 | 1.82E-09 | 0.97 | Appendicitis | 342,286 | 0.00007 | 24.93 | SCZ, ANX |
| rs17649913 | T | C | -0.06 | 0.01 | 9.14E-10 | 0.73 | Appendicitis | 342,286 | 0.00010 | 35.45 | SCZ, ANX |
| rs2595082 | G | T | -0.08 | 0.01 | 7.30E-16 | 0.74 | Appendicitis | 342,286 | 0.00020 | 69.50 | SCZ, ANX |
| rs3873387 | C | A | -0.06 | 0.01 | 1.51E-08 | 0.73 | Appendicitis | 342,286 | 0.00009 | 32.14 | SCZ, ANX |
| rs4666472 | G | A | 0.07 | 0.01 | 5.68-11 | 0.78 | Appendicitis | 342,286 | 0.00014 | 47.85 | SCZ, ANX |
| rs6842731 | A | T | 0.06 | 0.01 | 1.06E-11 | 0.35 | Appendicitis | 342,286 | 0.00012 | 40.44 | SCZ, ANX |
| rs7092009 | A | G | 0.05 | 0.01 | 4.78E-08 | 0.46 | Appendicitis | 342,286 | 0.00007 | 23.69 | SCZ, ANX |
| rs9935011 | A | G | -0.05 | 0.01 | 4.50E-08 | 0.71 | Appendicitis | 342,286 | 0.00009 | 29.36 | SCZ, ANX |
| rs994978 | T | C | -0.07 | 0.01 | 3.05E-14 | 0.43 | Appendicitis | 342,286 | 0.00014 | 46.49 | SCZ, ANX |
| rs6754306 | G | A | 0.07 | 0.01 | 3.37E-11 | 0.78 | Appendicitis | 342,286 | 0.00014 | 48.95 | BD |
| rs9273363 | C | A | 0.06 | 0.01 | 3.07E-10 | 0.72 | Appendicitis | 342,286 | 0.00011 | 37.80 | ANX |

**Table S4:** SNP list for exposure to appendectomy

| SNP | effect allele | other allele | beta | se | p-value | eaf | exposure | sample size | R2 | F | outcome |
| --- | --- | --- | --- | --- | --- | --- | --- | --- | --- | --- | --- |
| rs200540616 | C | T | 0.072785 | 0.011583 | 3.30E-10 | 0.783215 | Appendectomy | 342499 | 0.000114 | 38.97355 | MDD, SCZ |
| rs2584229 | T | C | -0.07722 | 0.012875 | 2.00E-09 | 0.824606 | Appendectomy | 342499 | 0.000104 | 35.5541 | MDD,BD |
| rs3866831 | G | A | -0.10299 | 0.009645 | 1.29E-26 | 0.469793 | Appendectomy | 342499 | 0.000331 | 113.2701 | MDD,BD, SCZ |
| rs2595110 | A | G | -0.07788 | 0.010352 | 5.33E-14 | 0.669536 | Appendectomy | 342499 | 0.000163 | 55.92972 | BD |
| rs4666472 | G | A | 0.071383 | 0.011528 | 5.93E-10 | 0.781908 | Appendectomy | 342499 | 0.00011 | 37.84128 | SCZ |

**Table S5:** Genetic correlation between exposure traits

| Trait 1 | Trait 2 | r_g_ | r_g_ se | r_g_ p-val | gcov |
| --- | --- | --- | --- | --- | --- |
| Tonsillectomy | Appendectomy | 0.36 | 0.09 | 0.0003 | 0.006 |
| Tonsillectomy | Appendicitis | 0.36 | 0.10 | 0.0002 | 0.007 |
| Tonsillectomy | MDD | -0.39 | 0.09 | 7.50E-05 | -0.01 |
| Tonsillectomy | SCZ | -0.13 | 0.05 | 0.01 | -0.01 |
| Tonsillectomy | BD | -0.10 | 0.05 | 0.03 | -0.01 |
| Tonsillectomy | ANX | -0.22 | 0.06 | 0.0006 | -0.006 |
| Appendectomy | Appendicitis | 1 | 0.03 | 1.10E-176 | 0.01 |
| Appendectomy | MDD | -0.32 | 0.14 | 0.02 | -0.005 |
| Appendectomy | SCZ | -0.04 | 0.07 | 0.52 | -0.002 |
| Appendectomy | BD | 0.02 | 0.05 | 0.65 | 0.0001 |
| Appendectomy | ANX | -0.0004 | 0.001 | 0.71 | -0.004 |
| Appendicitis | MDD | -0.14 | 0.13 | 0.25 | -0.002 |
| Appendicitis | SCZ | -0.0001 | 0.07 | 0.98 | -8E-06 |
| Appendicitis | BD | -0.03 | 0.05 | 0.58 | -0.001 |
| Appendicitis | ANX | -0.10 | 0.08 | 0.19 | -0.002 |

Abbreviations: r_g_: genetic correlation, se: standard error, gcov: genetic covariance
